# Resistomes and microbiome of meat trimmings and colon content from culled cows raised in conventional and organic production systems

**DOI:** 10.1186/s42523-022-00166-z

**Published:** 2022-03-10

**Authors:** Margaret D. Weinroth, Kevin M. Thomas, Enrique Doster, Amit Vikram, John W. Schmidt, Terrance M. Arthur, Tommy L. Wheeler, Jennifer K. Parker, Ayanna S. Hanes, Najla Alekoza, Cory Wolfe, Jessica L. Metcalf, Paul S. Morley, Keith E. Belk

**Affiliations:** 1 Department of Animal Sciences, Colorado State University, Fort Collins, CO USA; 2 Department of Microbiology Immunology and Pathology, Colorado State University, Fort Collins, CO USA; 3 U.S. Department of Agriculture, Agricultural Research Service, Roman L. Hruska U.S. Meat Animal Research Center, Clay Center, NE USA; 4 Department of Clinical Sciences, Colorado State University, Fort Collins, CO USA; 5 Veterinary Education, Research, and Outreach Program, Texas A&M University and West Texas A&M University, Canyon, TX 79105 USA; 6 Present Address: U.S. Department of Agriculture, Agricultural Research Service, U.S. National Poultry Research Center, Athens, GA USA; 7 Present Address: Intralytix, Columbia, MD 21046 USA

**Keywords:** Antibiotic resistance, 16S, Target-enriched metagenomics, Microbiome, Bovine

## Abstract

**Background:**

The potential to distribute bacteria resistant to antimicrobial drugs in the meat supply is a public health concern. Market cows make up a fifth of the U.S. beef produced but little is known about the entire population of bacteria (the microbiome) and entirety of all resistance genes (the resistome) that are found in this population. The objective of this study was to characterize and compare the resistomes and microbiome of beef, dairy, and organic dairy market cows at slaughter.

**Methods:**

Fifty-four (N = 54) composite samples of both colon content and meat trimmings rinsate samples were collected over six visits to two harvest facilities from cows raised in three different production systems: conventional beef, conventional dairy, and organic dairy (n = 3 samples per visit per production system). Metagenomic DNA obtained from samples were analyzed using target-enriched sequencing (resistome) and 16S rRNA gene sequencing (microbiome).

**Results:**

All colon content samples had at least one identifiable antimicrobial resistance gene (ARG), while 21 of the 54 meat trimmings samples harbored at least one identifiable ARGs. Tetracycline ARGs were the most abundant class in both colon content and carcass meat trimmings. The resistome found on carcass meat trimmings was not significantly different by production system (*P* = 0.84, R^2^ = 0.00) or harvest facility (*P* = 0.10, R^2^ = 0.09). However, the resistome of colon content differed (*P* = 0.01; R^2^ = 0.05) among production systems, but not among the harvest facilities (*P* = 0.41; R^2^ = 0.00). Amplicon sequencing revealed differences (*P* < 0.05) in microbial populations in both meat trimmings and colon content between harvest facilities but not production systems (*P* > 0.05).

**Conclusions:**

These data provide a baseline characterization of an important segment of the beef industry and highlight the effect that the production system where cattle are raised and the harvest facilities where an animal is processed can impact associated microbiome and resistomes.

**Supplementary Information:**

The online version contains supplementary material available at 10.1186/s42523-022-00166-z.

## Introduction

Public health officials have expressed ongoing concern regarding the potential influence of antimicrobial drug (AMD) exposures in food animals on the promotion and dissemination of antimicrobial resistant bacteria [1]. It is postulated that administering AMDs to food animals could indirectly result in resistant infections in humans [2–4]; this specific concern would be as a result of contamination of meat during the slaughter and harvest process by antibiotic resistant bacteria originating from the hide and gastrointestinal tracts of cattle. Recently, the Veterinary Feed Directive was enacted in the U.S. (U.S. Federal Registry 80 FR 31707) in efforts to reduce the use of medically important AMDs in food animal production.

Antimicrobial drugs are used to treat and prevent disease in food-producing animals [5]. Unlike other food-producing species where vertical integration is common, beef production is highly segmented and typically cattle are transferred/sold into multiple production operations by the time they are marketed for harvest. One beef industry sector where little research has been conducted regarding factors affecting antimicrobial resistance (AMR) is in ‘market cows’. Cows are mature female cattle, and ‘market cows’ are cattle that are culled from production of milk (dairy production) or production of beef calves (cow-calf production) following a decrease in productivity that makes their retention uneconomical. Market cows, when compared to ‘feedlot cattle’ (young cattle produced for meat that spend the last months of their lives in feedlots on high concentrate diets) have different diets and AMD exposures. Furthermore, market cows typically have a longer lifespan than feedlot cattle, with dairy cows living approximately 3–5 years and beef cows 8–10 years and feedlot cattle 15–24 months.

In beef cattle, it has been reported that 15.3% of cow-calf operations use AMDs for disease prevention [6], while 68% use AMDs to treat disease, although, on average, only 1.9% of cows within a herd are treated with AMDs [7]. According to the National Animal Health Monitoring System’s (NAHMS) 2007 to 2008 report, the most commonly used injectable AMD was tetracycline, which was used in 7.8% of operations. With the exception of non-cephalosporin beta-lactams, no other AMD was used in more than 1% of a beef-cow herd [7]. The 2014 NAHMS survey of dairy operations found that 91.3% used AMDs in at least a portion of cattle [8]. The most common reasons for treatment were mastitis, infections of the reproductive system, and infections causing lameness, which reportedly affected 22%, 7.7%, and 3.6% of cows within herds, respectively. For the treatment of mastitis, 63.2% of operations used cephalosporins as the primary AMD, while penicillin was the second most commonly used class of antimicrobial drug [8].

Antimicrobial drug administration in cow-calf and dairy operations is starkly different than in feedlot cattle, which commonly use AMDs by injection and in feed for the treatment and prevention of infectious diseases. Studies conducted by the USDA have found that that 71.2% of cattle in feedlots with a capacity above 1000 animals received a macrolide AMD in feed, while 3.7% or 13.8% of cattle (with a lower occurrence in older cattle) received injectable macrolides or fluoroquinolones for treatment or prevention of infectious respiratory diseases [9].

The majority of market cows are raised in a production system that uses AMDs for health and welfare of animals (referred to here as conventional production), but production systems that restrict use of AMDs are a growing segment of the dairy and beef industries. In 2013, organic dairies produced 2.2 million pounds of milk, which accounted for 4.4% of the total milk produced in the U.S.—a market share that continues to increase annually [10]. It is postulated that one reason for the increase in market share for organic dairy products is the perception that restricting AMD exposures decreases selection pressure for AMR.

Investigations into how AMD exposures influence the ecology of all bacteria (the microbiome) and all resistance genes (the resistome) of feedlot cattle and milking dairy cows have traditionally employed culture-dependent approaches, and more recently quantitative PCR (qPCR), and shotgun sequencing methods [11–15]. However, the literature regarding the effect of antibiotic use on AMR in market cows is sparse [16] when compared to that of fed cattle [14, 17, 18]. This is a critical data gap as market cows and bulls comprise 17–19% of beef cattle harvested annually at federally inspected slaughter facilities in the U.S. slaughter [19, 20]. Therefore, it is important to improve the baseline understanding of AMR in beef produced from market cows to better understand the AMR risk associated with beef production in the U.S.

Metagenomic investigations provide a more complete understanding of microbial ecologies, and target-enriched sequencing approaches provide a more efficient means for interrogating the resistome ecology by targeting specific genes and reducing sequencing of background DNA [21]. Hence, the objective of this study was to use target-enriched metagenomic sequencing and 16S rRNA gene sequencing to characterize the resistomes and microbiome of colon content and trimmings that were collected from the carcasses of beef, dairy, and organic dairy market cows at harvest and compare them to each other.

## Materials and methods

### Description of the study population and sampling strategy

Cattle that were sampled as part of this study were market cows that had been presented for slaughter at a participating harvest facility. Cows from three production systems were sampled in this study: conventional beef cows ‘CON-B’, conventional dairy cows ‘CON-D’, and organic dairy cows ‘ORG-D.’ Due to the nature of beef cattle production, it was difficult to identify significant numbers of culled beef cows that were raised without AMD exposures thus this group was not included in this study. While AMD exposures are permitted in CON-B and CON-D cows, all food animals that have been treated with AMDs must undergo a mandatory withdrawal period prior to harvest to ensure that there are no antibiotic residues in the tissues. ORG-D cows were raised on certified organic dairy operations until slaughter. Records regarding health and history of treatment of study cattle with AMDs were not available. Colon content samples were assumed to reflect influences of the production environment, including AMD exposures, while meat trimmings samples were assumed to reflect the potential for AMR bacteria to enter the food supply, and thus were considered to be a potential indicator of human public health risk.

### Processing facility overview

**Fig. 1:**
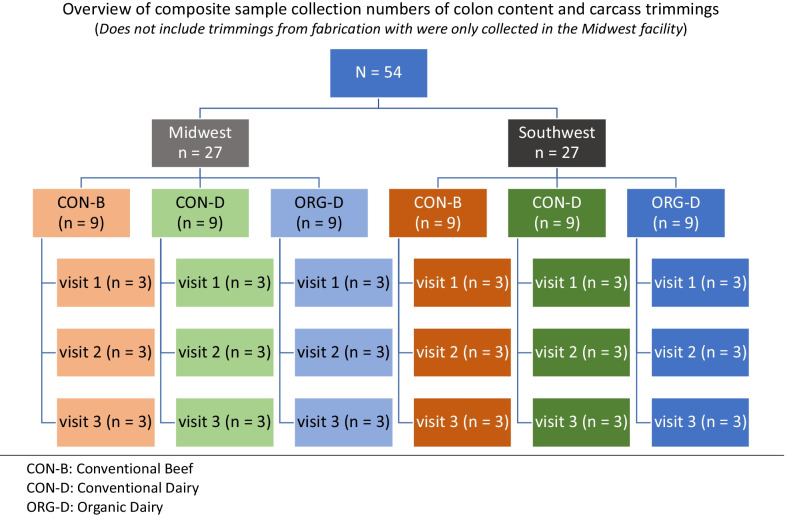
Overview of samples collected during each of the six visits to the processing facility. Besides samples collected from fabrication, which only occurred at the Midwest plant, each of the visits resulted in the same number of composite samples

Samples were collected during six visits over a six-month period to two U.S. commercial beef packing facilities that harvested market cows (Fig. 1, Additional file 2: Data 1). Each plant was visited three times as an attempt to account for seasonal variability. One harvest facility was in the Southwest while the other was in the Midwestern U.S. Samples were collected from cow carcasses generated from all three production backgrounds at each plant location (CON-B, CON-D, and ORG-D). At each of the six visits to the processing facilities, nine composite colon content samples and nine composite carcass meat trimmings samples were collected: three from each production system/sample type (54 composite samples = 2 facilities × 3 visits per facility × 3 systems × 3 replicates). At the three Midwest plant visits, in addition to the trimmings from the chilling cooler, six composite meat trimmings from fabrication were collected: three from conventional lots and three from organic (18 composite samples = 1 facility × 3 visits per facility × 2 systems × 3 replicates). The CON-B and CON-D cattle were identified by phenotype (with visual inspection of hide on cattle structure and hide coloring) while ORG-D cattle were identified through verification of organic certification and were subsequently followed through the harvesting process.

### Colon content sampling

The 54 composite colon content samples, comprised of total 535 individual animals in composites of nine to ten individual colons (54 composite samples = 2 facilities × 3 visits per facility × 3 systems × 3 replicates). During each harvest facility visit, three composite samples were obtained from each production system, for a total of nine composites per plant per visit. Individual colon content samples were acquired by obtaining a portion of the sigmoid colon from the evisceration belt by making an incision in the colon and transferring approximately 25 g of content into a plastic bag. Samples were stored at 4 °C and shipped overnight to the US Meat Animal Research Center (USMARC; Clay Center, NE) for compositing immediately upon arrival. Composite samples were created by combining 5 to 5.5 g from nine to ten individual colons (50 g total/sample) from within one production system (Additional file 2: Data 1). Composited samples were shipped on ice overnight to Colorado State University (CSU; Fort Collins, CO) and stored at -80 °C until further processing.

### Carcass meat trimmings collected in chilling coolers

Fifty-four composite carcass meat trimmings samples, from 532 individual carcasses, (approximately 900 g each) were obtained from carcasses located in the plants’ chilling coolers 24 h ± 4 h after colon content samples were collected (54 composite samples = 2 facilities × 3 visits per facility × 3 systems × 3 replicates). While meat trimmings were collected from the same defined production backgrounds from the previous day’s harvest, the same carcasses were not necessarily used for both sample types. Approximately 90 to 130 g of meat trimmings (comprised of a combination of the brachiocephalicus, trapezius, rhomboideus, and splenius muscles) from seven to ten carcasses were excised and combined to create three composite samples per production system (Additional file 2: Data 1). Samples were immediately placed on ice and transported to CSU for processing within 24 h of collection.

### Final meat trimmings collected during fabrication

The Southwest facility did not have a certified organic labeling program, so it was not possible to maintain carcass identity to the completion of fabrication (carcass disassembly). Therefore, final meat trimmings from the final stages of fabrication were only collected in the Midwest facility. Furthermore, because CON-D and CON-B cows were not marketed separately, the only comparison that could be made was between conventional and organic production systems. During each of the three visits to the Midwest facility, three organic and three conventional final meat trimmings samples were obtained for a total of 18 composite samples (18 composite samples = 1 facility × 3 visits per facility × 2 systems × 3 replicates). Each composite final meat trimmings sample was approximately 900 g, though because of fabrication it is not known how many individual animals this represented. Samples were immediately placed on ice and transported to CSU for processing within 24 h of collection.

### DNA Isolation for sequencing

Upon arrival at CSU, carcass and final meat trimmings were processed to collect rinsates within 24 h. To each sample bag, 180 mL of phosphate-buffered saline was added and the bag was hand-massaged. After massaging, all supernatant was centrifuged (10,000 × g for 10 min at 4 °C) to pellet the cells. Pellets were stored at -80 °C until DNA isolation. DNA from each thawed pellet was extracted using the QIAamp PowerFecal DNA Kit (Qiagen, Hilden, Germany) following the manufacturer’s instructions. Colon content was thawed at 4 °C prior to DNA isolation; a 0.2 g aliquot of each composite colon sample was used in the QIAamp PowerFecal DNA Kit (Qiagen, Hilden, Germany) using the manufacturer’s instructions for DNA extraction. Along with DNA extraction of samples, three molecular water samples were extracted at the same time using the same methods to act as extraction controls. When necessary, DNA was concentrated using ethanol precipitation.

### Library preparation and sequencing

#### Target-enriched shotgun metagenomic sequencing

Target enriched metagenomic sequencing libraries were prepared using the customized SureSelectXT-HS system (Agilent Technologies, Santa Clara, CA) to target determinants of AMR for 5557 gene accessions using methods previously described [21]. Target-enriched sequencing was conducted at the University of Colorado Denver Genomics and Microarray Core Facility (2 × 150 bp sequencing on the Illumina NovaSEQ 6000, Illumina, Inc., San Diego, CA) with a target depth of 20 million reads per colon content sample and 100 million reads for all rinsate samples. Additionally, two ZymoBIOMICS mock communities (Zymo Research, Irvine, CA) with meta-sequin (one with Mix A and one with Mix B) spiked in at 2% of the mock community DNA by weight [22], were sequenced on each lane for normalization during the downstream analysis.

#### 16S rRNA gene sequencing

Aliquots of DNA extracted from all samples were shipped to Novogene Corporation (Sacramento, CA) for 16S rDNA library preparation and sequencing. The V4 region of the 16S ribosomal subunit was amplified using the 515-806R primer set. Paired-end sequencing (2 × 250) was conducted on an Illumina HiSeq 2500 (Illumina, Inc., San Diego, CA) with a target of at least 100,000 reads per sample (excluding extraction controls).

### Bioinformatics

#### Target-enriched shotgun metagenomic sequencing

Sequencing data were processed using the AMRplusplus [23] pipeline, with modifications. Briefly, sequencing reads were trimmed using Trimmomatic [24] and host DNA was removed using BWA [25]. Duplicate reads (reads that were identical for the entire length of the read), were removed via BBTools’ dedupe script (https://jgi.doe.gov/data-and-tools/bbtools/). BWA was used to align non-host reads to the MEGARes database to classify reads for AMR gene accessions [23]. Hits for gene accessions that require a single nucleotide polymorphism to confer resistance, as identified by the MEGARes database [23], were removed from the downstream analysis. Relative abundance of AMR genes was defined as the total number of reads belonging to one classification (such as class) as a proportion of the total number of AMR reads within a sample.

#### Normalization of counts and correction for lane and false positive rates

For normalization on each count table the same process was followed: raw data was CSS normalized via ‘cumNorm’ function in MetagenomeSeq, CSS tables were corrected for lane effect, and then based on the mock communities a false positive threshold was set and subtracted from all counts.

##### Accounting for lane effect

In addition to sequencing samples across different lanes and runs of a NovaSEQ 6000, each lane of samples that was sequenced also had two mock communities (ZymoBIOMICS) of known DNA quantity and 2% meta sequins by DNA molecular weight (Either Mix A or B) on each lane. Upon shotgun sequencing, the resulting mock community/sequin samples were aligned to a FASTA file of the 86 known meta sequin (https://s3.amazonaws.com/sequins/annotations/Metasequins_details.txt) using bwa-mem [25]. Samtools [26] was then used to covert the SAM files to BAM files, sort and index them and count alignment numbers with ‘idxstats.’ From the alignment numbers, total sequins numbers were calculated for each lane and each lane was normalized based on this number to counter lane effect.

##### Correcting for false positives in AMR data with a mock community

The ZymoBIOMICS mock community, composed of ten known organisms was processed in the same way as other samples in the study (library preparation with a custom library bait capture system specific to AMR genes). After sequencing, the mock communities AMR counts were generated in the same way as described with modification to the AMRplusplus pipeline. To create a list of what AMR genes were present in the 10 organisms included in the mock community, draft complete genomes FASTA files were obtained from NCBI. The bbmap script ‘randomreads.sh’ was used to fragment the genomes (2 × 150) with 500,000 fragments per genome. From there, the synthetic reads were aligned to the MEGARes database [23] using bwa-mem [25]. Samtools [26] was then used to covert the SAM files to BAM files, sort and index them and count alignment numbers with ‘idxstats.’ The results count numbers were converted to a presence/absence count table and aggregated across all genomes for one composite count for the mock community.

The known counts for the synthetic reads were compared to the CSS normalized counts that were generated through sequencing. The CSS mock community lowest count that also had the presents of a synthetic DNA reads was established as the cutoff point. From there, the CSS normalized cutoff number threshold was subtracted from every count in the count table at the gene level. Any value that was below zero was treated as a zero and any gene that had zero hits across all samples as a result of this action were removed from the count table.

#### 16S rRNA gene sequencing

Demultiplexed samples were obtained from Novogene and processed with QIIME2 v. 2018.11 [27]. Files were imported into QIIME2 using the ‘qiime tools import’ command using the paired end option. Exact sequence variants were assigned via DADA2 [28] with the first 20 nucleotides of both the forward and reverse reads trimmed, as well as truncation at nucleotide 220 of the forward reads and 230 on the reverse reads. Phylogenetic trees were generated using MAFFT v. 7 [29] and FastTree2 [30]. Taxonomic classification was conducted using a Naïve Bayesian classifier pretrained using the 515-806R primers on the Greengenes database [31]. Reads that were assigned to chloroplasts and mitochondria and those that did not have a kingdom classification were removed. After negative controls were assessed, they were removed and tables were parsed by sample type.

### Statistical analysis

#### Experimental design

The study was constructed as a 2 × 3 factorial, where time of collection was treated as random, the first fixed factor was facility (Midwest or Southwest), and the second fixed factor was cow production background (CON-B, CON-D, or ORG-D). Due to the uncertainty surrounding the geographic distribution of cattle feeding into each facility, only differences in actual facility, not region of harvest, could be made. Type 1 errors were established at α = 0.05 with trends considered between 0.05 and 0.1.

#### Target-enriched shotgun metagenomic sequencing

Shannon’s diversity was evaluated using the ‘car’ (v. 2.1-6) and ‘emmeans’ (v. 1.1) packages in R (version 3.4.2) via the ‘Anova’ and ‘lsmeans’ functions, respectively. Mean separation was accomplished using the ‘pairs’ function of the ‘emmeans’ R package. Non-metric multidimensional scaling ordination was performed using the Hellinger transformation and Euclidean distances in the metaMDS function of Vegan [32], with differences compared using PERMANOVA using Vegan’s function ‘adonis’. Within these results, an R^2^ close to 1 indicates dissimilarity between groups while an R^2^ value closer 0 suggests more evenness between groups and an R^2^ < 0 indicates greater differences within groups than between groups. Log_2_ fold changes were calculated using the ‘FitZig’ function in metagenomeSeq [33] by fitting multivariate zero-inflated Gaussian mixture models. Limma’s ‘makecontrast’ function [34] was used for pairwise mean separation and adjusted with the Benjamini–Hochberg procedure [35], with only classes/mechanisms of resistance above 1.0 log base 2 average relative abundance considered biologically relevant. Classes and mechanisms associated with beta-lactam and tetracycline resistance were specifically noted in comparisons given their wide use in production and VolcaNoseR [36] was used for pairwise volcano plot visualizations of these comparisons.

#### 16S rRNA gene sequencing

Differences in read numbers were assessed with the ‘anova’ and ‘pairs’ functions from base R and emmeans, respectively. Tables were rarified as follows: trimmings collected from the cooler at 48,584 reads, trimmings collected from fabrication at 50,684, and colon content at 92,539 reads; each sample type was rarefied to the lowest number of reads within a sample type that allowed for the retention of all samples. Alpha diversity was calculated on a rarified table using Faith’s phylogenetic diversity. Beta diversity was assessed using weighted and unweighted UniFrac distances. Differences in alpha diversity were assessed with Kruskal–Wallis tests, while beta diversity differences were evaluated using PERMANOVA using Vegan’s function ‘adonis’ for interactions and PERMANOVA with PERMDISP (999 permutations, performed in QIIME2 v. 2021.4) for main effects and pairwise comparisons. Differential abundance was calculated with the ‘qiime composition’ tool using ANCOM [37] on phyla, class, order, family, and genus of the total microbiome (ASV level was not considered due to the sparsity of these counts). UniFrac distances were visualized using principal coordinates analysis plots generated in EMPeror [38].

### Data availability

Raw sequence reads for all samples described in this project have been deposited the NCBI BioProject PRJNA736075.

## Results

This study was designed as a 2 × 3 factorial evaluating two facilities and three production systems and their interaction. When the statistical interaction between facilities and production systems was evaluated in each model, no significant interactions (*P* > 0.05) were observed in any model, i.e., facility and production system variables independently acted on the resistomes and microbiome of all sample types. As a result, only main effects are presented.

### Target-enriched shotgun metagenomic sequencing

A total of 892,717 reads were classified to AMR gene accessions. All colon content samples (54/54) contained reads classified to at least one AMR gene accessions. On the other hand, 21/54 (Table 1) of the trimmings from the coolers and 5/18 of the trimmings collected from fabrication contained sequences that aligned to at least one AMR gene accessions.

**Table 1:** Carcass meat trimmings rinsates that had at least one hit attributed to a determinant of antimicrobial resistance (positive samples/total samples tested)

Region	Conventional beef	Conventional dairy	Organic dairy	Total
Midwest	2/9	3/9	4/9	9/27
Southwest	4/9	4/9	4/9	12/27
Total	6/18	7/18	8/18	21/54

Colon content contained more (*P* < 0.001) raw reads classified to AMR gene accessions (mean = 15,151, range 5457 to 31,756) than carcass meat trimmings (mean = 2798, range 0 to 17,735) or final meat trimmings (mean = 3152, range 0 to 4677). There was no significant difference (*P* = 0.99) between the two types of meat trimmings in raw read count. Within colon content, the numbers of raw reads classified to AMR gene accessions did not differ (*P* = 0.25) by facility. However, between production systems, ORG-D had fewer (*P* = 0.001, Table 2) AMR raw reads (mean = 12,018) than CON-D (mean = 14,471) or CON-B (mean = 18,967).

**Table 2:** Average number of raw reads aligning to determinants of antimicrobial resistance in colon content samples, by production system and facility

Sample Origin	Midwest Mean (range)	Southwest Mean (range)	Overall average Mean (range)
Conventional beef	20,274	17,659	18,967
	(12,265 to 31,756)^a^	(9,502 to 25,337)	(9,502 to 31,756)
Conventional dairy	17,521	11,421	14,471
	(12,532 to 28,002)	(5606 to 15,930)	(5606 to 28,002)
Organic dairy	13,088	10,947	12,018
	(8479 to 22,314)	(5457 to 17,241)	(5457 to 22,314)
Overall average	16,961	13,342	15,152
	(8479 to 31,756)	(5457 to 25,337)	(5457 to 31,756)

**Fig. 2:**
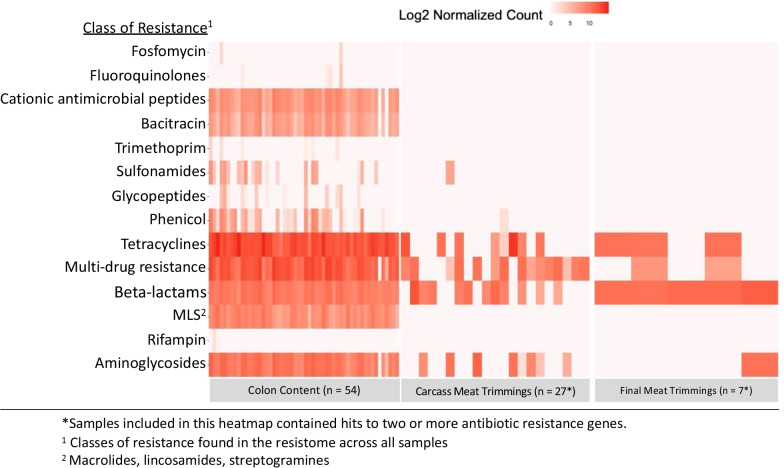
Log_2_ abundance of sequencing reads aligning to determinants of AMR found in more than 1% of the resistome, by drug class and sample type

Considering the relative abundance of reads classified to AMR gene accessions, the resistomes of all sample types were dominated (94–100% of the resistome) by four classes of antibiotic resistance (Fig. 2): tetracycline, aminoglycosides, beta-lactams, and mechanisms that conferred resistance to more than one class of resistance (e.g., multi-drug efflux pumps). The resistome of carcass meat trimmings was comprised of determinants encoding resistance to tetracycline (45%), beta-lactams (22%), multi-drug mechanisms (22%), and aminoglycoside (11%). At the mechanism level, the most abundant classification for the resistome of carcass meat trimmings were tetracycline resistance ribosomal protection proteins (39%), class A beta-lactamases (22%), and multidrug efflux pumps (16%). The resistome of final meat trimmings contained the highest relative abundance of reads aligning to determinants of beta-lactam (65%) and tetracycline resistance (25%), followed by resistance determinants of aminoglycosides and multi-drug mechanisms, respectively; class A beta-lactamase (57%) and tetracycline resistance major facilitator superfamily MFS efflux pumps (16%) were the most abundant mechanisms of resistance. Finally, colon content contained a resistome comprised of determinants for tetracycline resistance (50%) and resistance to multiple drug classes (30%). Resistance to aminoglycoside, beta-lactams and MLS all comprised 3–8% of the resistome. At the mechanism level, colon content was comprised of 48% tetracycline resistance ribosomal protection proteins, 19% multidrug efflux pumps, and 10% multidrug regulators.

#### Meat trimmings rinsate resistomes

**Fig. 3:**
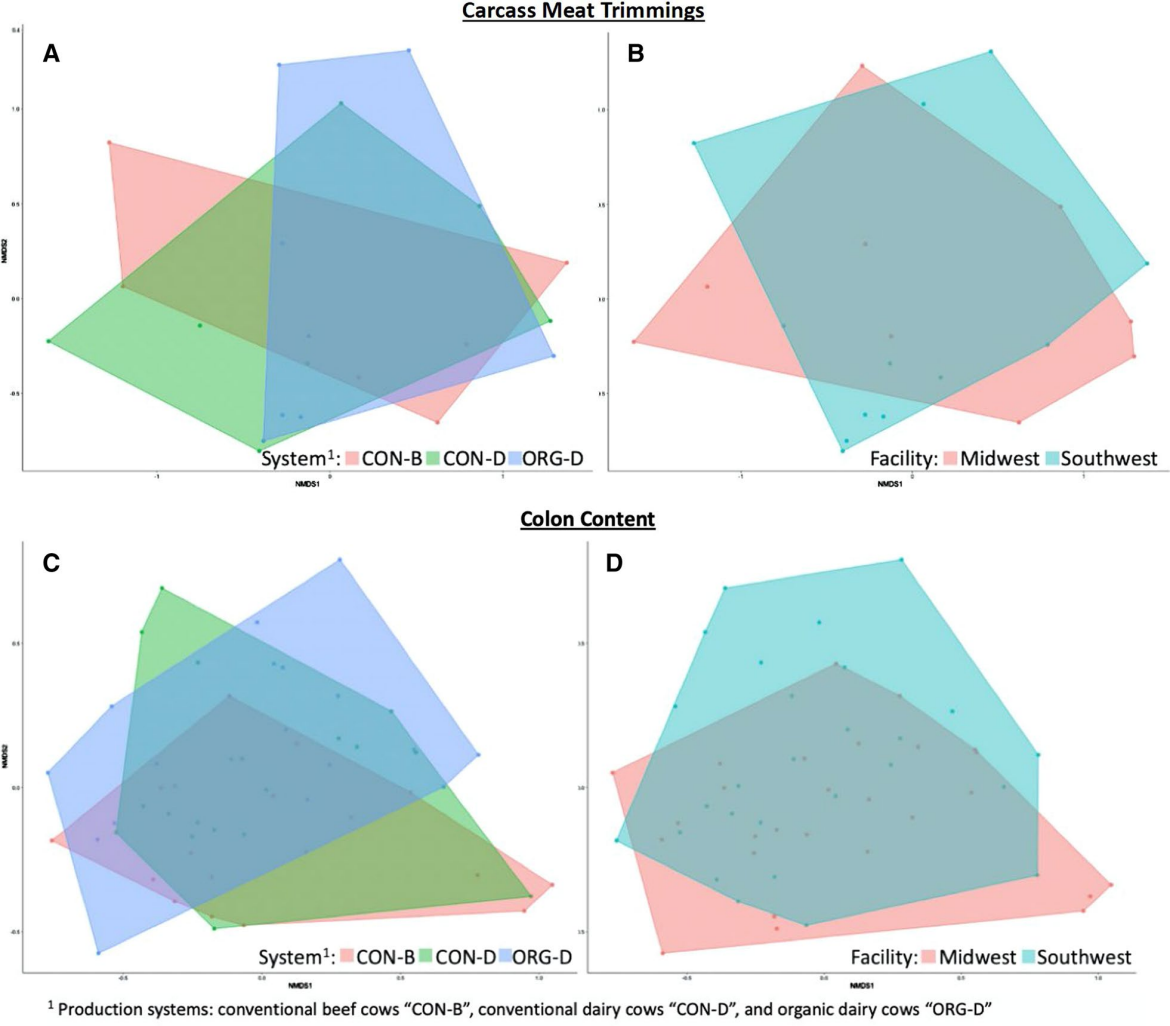
Ordination of sequencing reads aligning to determinants of AMR for carcass meat trimmings rinsates samples obtained in the chilling cooler and colon content, by production practice and harvest facility. Non-metric multidimensional scaling of Euclidean distances revealed no difference between (**A**) carcass meat trimmings of production practices (*P* = 0.84, R^2^ = 0.00) or (**B**) carcass meat trimmings of facility of harvest (*P* = 0.10, R^2^ = 0.09). On the other hand, ordination of resistome colon content revealed that the (**C**) production system had an effect (*P* = 0.01; R^2^ = 0.05) on the resistome composition, although (**D**) harvest facility did not (*P* = 0.41; R^2^ = 0.00)

Carcass meat trimmings did not differ (*P* > 0.05) in Shannon’s diversity of AMR genes between production system or facility. Resistome composition of carcass meat trimmings, as measured by Euclidian distances, also did not differ (production system *P* = 0.84, R^2^ = 0.00; facility of harvest *P* = 0.10, R^2^ = 0.09) between the production system or facility (Fig. 3A, B), though differences between facility were considered a trend. When the relative abundance of AMR gene accession hits at the AMR class level were compared between production systems (Additional file 2: Data 2) and facility (Additional file 2: Data 3) for carcass meat trimmings, aminoglycoside resistance was higher (*P* < 0.05) in the Midwest. Also, of note was the spareness of carcass meat trimmings sample hits associated with all four primary classes of resistance identified, with only one sample containing hits associated with all four AMR classes (Additional file 2: Data 4). At the mechanism level (Additional file 2: Data 5 and 6), when resistance to tetracycline and beta-lactam were specifically evaluated, only class A beta-lactamases had counts in more than half the samples (Additional file 2: Data 7); determinants of class A beta-lactam resistance had more (*P* < 0.05) normalized counts in CON-B trimmings when compared to both CON-D and ORG-D (3.8 and 3.4 log_2_ fold difference in abundance, respectively), and there was no difference (*P* > 0.05) in abundance between carcass meat trimmings from dairy cattle (CON-D vs. ORG-D).

Shannon’s diversity and community level ordination were not formally compared in final meat trimmings due to small sample sizes (after the removal of samples with reads aligned to only one gene, only five samples remained—three from conventional production and two from organic).

#### Colon content resistomes

Ordination of Euclidian distances revealed that the production system cows were raised in had an effect (*P* = 0.01; R^2^ = 0.05, (Fig. 3C)) on the resistome composition, while harvest facility did not (*P* = 0.41; R^2^ = 0.00 (Fig. 3D)). Shannon’s diversity of AMR gene determinants in colon content did not differ (*P* > 0.05) between harvest facility or production system. Classes of resistance that differed between harvest facility (such as resistance to phenicol, sulfonamides, glycopeptides, fluoroquinolones, trimethoprim, fosfomycin and rifampin) were all present in less than 3% of the total resistome (Additional file 2: Data 8). Between production systems (Additional file 2: Data 9), beta-lactam resistance was higher (*P* = 0.02) in CON-B than ORG-D; with the only other differences observed between AMR classes that comprised less than 3% of the total resistome.

**Fig. 4:**
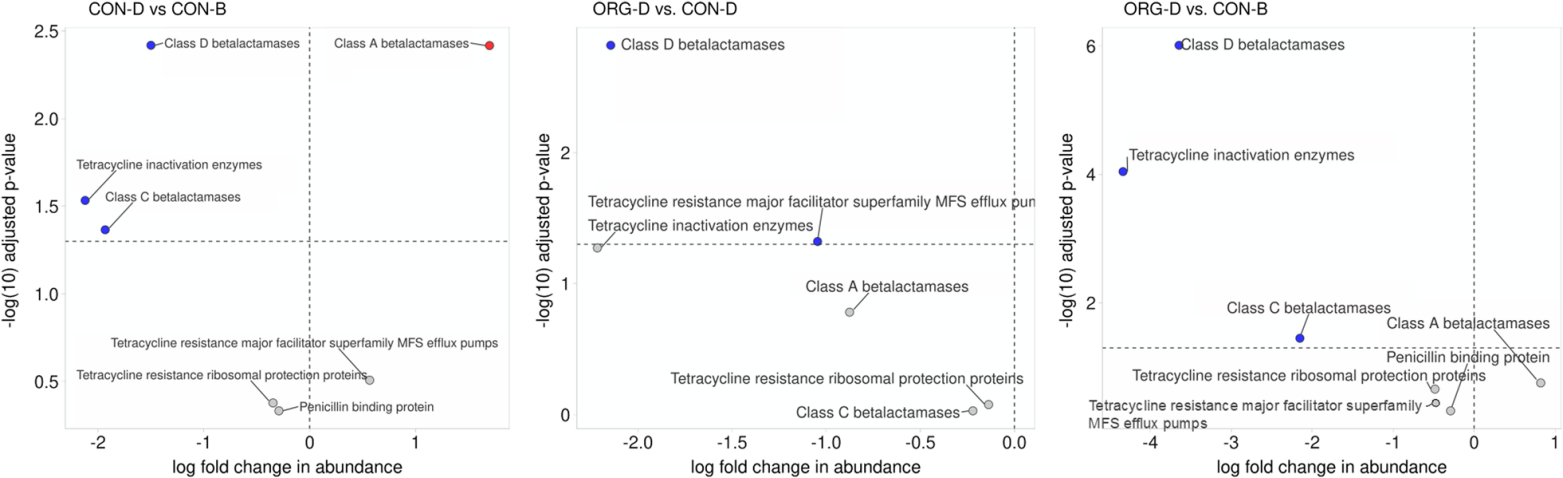
Pairwise comparisons of differences in log_2_ fold change in relative abundance of colon content antibiotic resistance determinant mechanisms of resistance specific to tetracycline and beta-lactams resistance between different production system (CON-D = Conventional Dairy, CON-B = Conventional Beef, and ORG-D = Organic Dairy). The -log(10) adjusted p-value critical value was 1.3 which is equivalent to α = 0.05. A blue dot indicates the first production system abundance is significantly lower than the second, a red dot indicates first production system abundance is significantly higher than the second, and a gray dot indicates no significant difference between the production systems

When mechanisms of resistance specific to tetracycline and beta-lactam resistance were compared between production system in the colon content, several mechanisms differed (*P* < 0.05) between pairwise comparisons (Fig. 4). Between facilities, class A beta-lactamase resistance was higher (*P* = 0.04) in the Midwest than the Southwest. When considered separately, both production system and facility contained other mechanisms that differed between groups (Additional file 2: Data 10 for facility and Additional file 2: Data 11 for production system).

### 16S rRNA gene sequencing

**Fig. 5:**
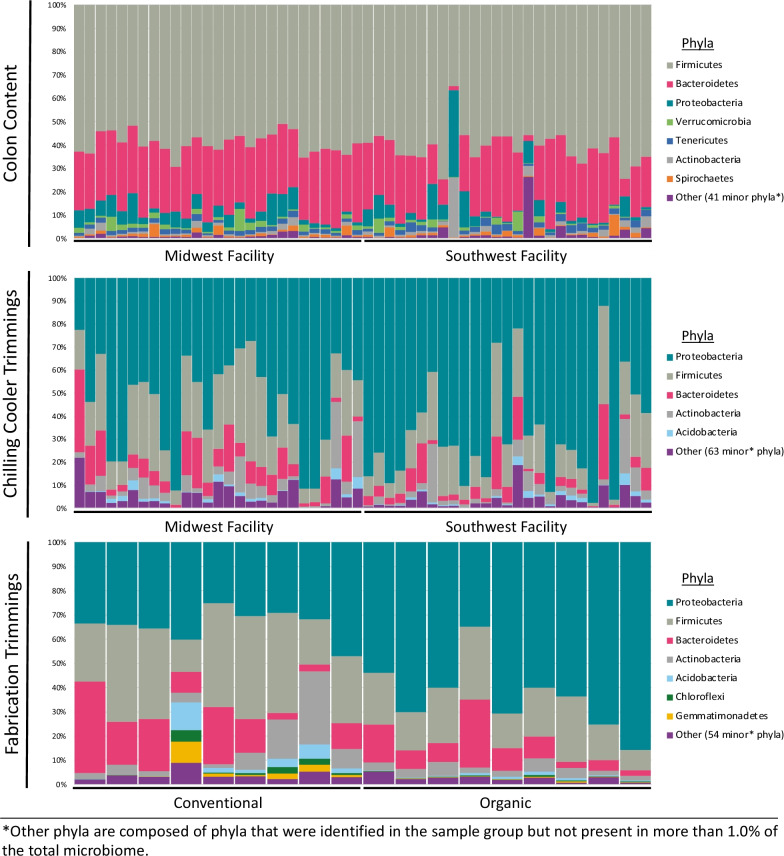
Relative taxonomic abundance at the phyla level for colon content (top), carcass meat trimmings (middle), and final meat trimmings (bottom)

After quality filtering, 16,926,768 reads were retained for microbiome analysis (average = 132,240, range 41,528 to 194,100). Proteobacteria (63%), Firmicutes (19%) and Bacteroidetes (7%) phyla were present in the highest relative abundance in the overall microbiome of carcass meat trimmings (Fig. 5); within Proteobacteria, Gammaproteobacteria was the most common with Pseudomonadales and Enterobacteriales in the highest abundance at the order level (as well as a large portion that classified to Gammaproteobacteria but not beyond that). Similarly, the microbiome of final meat trimmings were mainly comprised of Proteobacteria (49%), Firmicutes (25%) and Bacteroidetes (10%) (Fig. 5); though the highest abundance family in the final meat trimmings were Moraxellaceae. In contrast to the meat trimmings samples, the colon content microbiome was comprised primarily of Firmicutes (60%) followed by Bacteroidetes (27%). Proteobacteria, Tenericutes, Verrucomicrobia, Actinobacteria and Spirochaetes made up between 1 to 5% of the total microbiome in the colon samples (Fig. 5). Within Firmicutes, the order Clostridiales was the most common comprised of Ruminococcaceae, Lachnospiraceae and unclassified at the family level bacteria; the phyla Bacteroidetes was comprised of the highest abundance of Bacteroidales (data not shown).

**Fig. 6:**
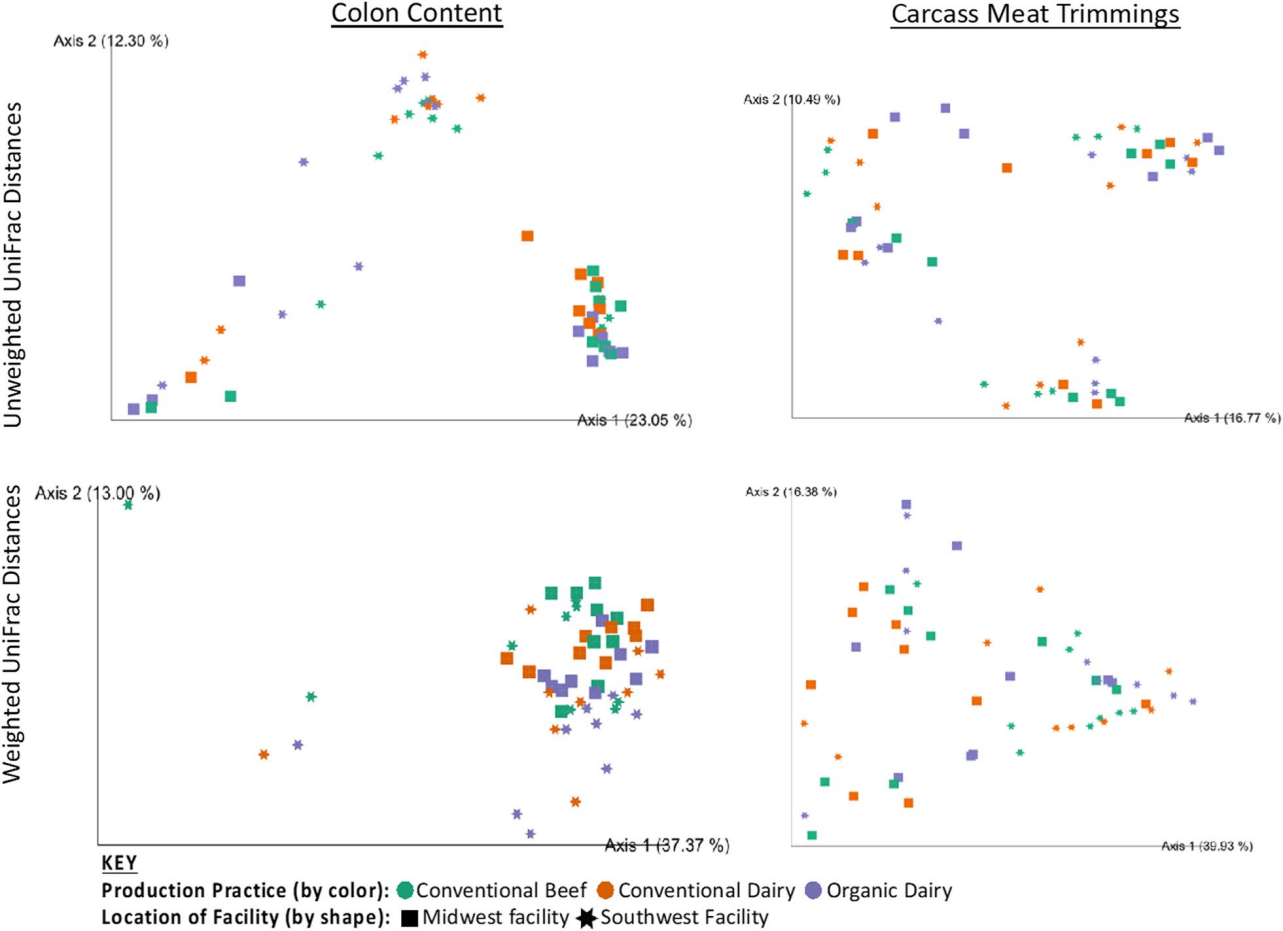
Beta diversity of colon content and carcass meat trimmings (as measured by weighted and unweighted UniFrac distances and compared with PERMANOVA). Samples are colored by the production practice the cows were raised in. Shapes correspond to the facility where the cows were slaughtered. In carcass meat trimmings, weighted UniFrac distances were different (*P* = 0.005) between harvest facilities (although unweighted distances were not different (*P* = 0.646)). There was no difference (*P* > 0.05) between the production facility where the cows were raised. For colon content, harvest facilities had different (*P* < 0.05) microbiome, but not production-system weighted UniFrac distances (*P* > 0.05)

Alpha diversity of carcass meat trimmings did not differ by cow production system (*P* = 0.98) or facility location (*P* = 0.28). Weighted UniFrac distances of carcass meat trimmings differed (PERMANOVA *P* = 0.005, PERMDISP *P* = 0.138) by facility, yet unweighted UniFrac did not (PERMANOVA *P* = 0.65, PERMDISP *P* = 0.59 [Fig. 6]). Moreover, the production system the cows were raised in did not affect beta diversity (weighted UniFrac PERMANOVA *P* = 0.64, PERMDISP *P* = 0.19; unweighted UniFrac PERMANOVA *P* = 0.87, PERMDISP *P* = 0.06 [Fig. 6]). When log-fold change was assessed in carcass meat trimmings at the phyla, class, order, family and genus levels, there were no differences (*P* > 0.05) between production systems though there were some differences (*P* < 0.05) between harvest facilities (Additional file 1: Figure S1).

Alpha diversity of colon content was different (*P* = 0.01) between facilities, but not between production systems (*P* = 0.39). Colon content beta diversity differed (weighted UniFrac PERMANOVA *P* = 0.001, PERMDISP *P* = 0.001; unweighted UniFrac PERMANOVA *P* = 0.00, PERMDISP *P* = 0.15 [Fig. 6]) by facility, while the production system the cows were raised in did not (weighted UniFrac PERMANOVA *P* = 0.09, PERMDISP *P* = 0.97; unweighted UniFrac PERMANOVA *P* = 0.22, PERMDISP *P* = 0.15 (Fig. 6)). At the phyla, class, order, and family levels feature comparison between groups using ANCOM found no microbiome differences (*P* > 0.05) among production system in the colon content. At the genus level, Psychrobacter was higher (*P* < 0.05) in CON-B and CON-D when compared to ORG-D. Among facilities, there were differences (*P* < 0.05, ANCOM) between some bacteria at different taxonomic classification level (Additional file 1: Figure S1).

In final meat trimmings obtained from the Midwestern facility, those from conventional cows had a higher alpha diversity (*P* = 0.04; [Additional file 1: Figure S2]) and different beta diversities than those of cows raised in organic management systems (weighted UniFrac PERMANOVA *P* = 0.003, PERMDISP *P* = 0.02; unweighted UniFrac PERMANOVA *P* = 0.07; PERMDISP *P* = 0.006; [Additional file 1: Figure S2]). While there were no phyla, class, or order level differences (*P* > 0.05, ANCOM) between conventional and organic final meat trimmings; the genus Azomonas from the family Psychromonadaceae were found to be higher (*P* < 0.05; W = 1461) in organic final meat trimmings.

## Discussion

The target-enriched metagenomic data presented here indicated that production system and facility of harvest had minimal effects on the composition of the resistome found on meat trimmings prior to distribution in the food chain. These data, along with the fact that colon content resistome differed between production systems, suggest that a combination of animal production system and the harvest facility may influence the composition of the end-product resistomes. Previous studies of the microbiome and resistome of beef production have focused on fed-beef or lactating dairy cows [13–15, 39], thus there is limited data related to the AMR of market cows; though recently we have published culture and AMR qPCR data on the same cattle included in this study [19].

Not unexpectedly, there was a greater raw number of reads classified as AMR determinants in colon content samples compared to meat trimmings, this was attributed to much higher microbial load in the colon content. While expected, this finding is important in the interpretation of this study as both phyla of microbiome and class of AMR gene determinants are reported in relative abundance to the total reads within the microbiome or resistome and not absolute numbers. As a result, even if relative abundance in colon-content and meat trimmings is similar, the absolute number of read copies is magnitudes higher colon content due to the higher biomass. While this study found tetracycline to be the most abundant class of resistance on carcass meat; similar target-enriched sequencing methods used to investigate meat trimmings collected from fed beef cattle, found beta-lactams to be the most abundant class of resistance [40]. In contrast, a study on ground beef in retail stores found tetracycline to be the predominate class of resistance [41]. Culture-based investigations from the same sampling as this study but separately published [19] detected tetracycline-resistant *E. coli* on 6.7% of carcass meat trimmings and 20% of the final meat trimmings. The low percentage of tetracycline-resistance in *E. coli* from culture work demonstrates one benefit of the culture-free approach taken here, as this study includes tetracycline-resistance not limited to one indicator organism. This is essential when looking at the microbial ecology of AMR genes as resistant genes can reside in environmental commensal bacteria but transfer to pathogens over time [42, 43], as a result understanding a resistant gene within an entire bacterial community can give a more complete picture than just one indicator organism. Past work in feedlot feces has found aminoglycoside resistance to increase over the cattle feeding period in the absence of direct drug exposure [14, 44]. Therefore, a greater amount of aminoglycoside resistance on the meat trimmings from the Midwestern facility could be an indicator of environmental factors rather than direct drug exposure, such as geographical region of feeding [18].

Within colon content, across both facilities and all production systems, tetracycline resistance determinants accounted for half of all hits to AMR genes; this was not unexpected as several studies have documented similar findings in colon contents of beef cattle [13], as well as in feces for both dairy [15] and feedlot cattle [14]. The qPCR results from the same sampling scheme but published separately [19] also saw high levels of tetracycline AMR genes (61 to 96% detection of three different genes) as well as beta-lactam AMR genes detection in the colon content. The high relative abundance of hits associated with determinants of multidrug resistance mechanisms in colon contents is surprising as a past study in feedlot cattle found this mechanism in much lower levels using similar techniques [44]. The mechanisms that made up hits associated with multi-drug resistance included efflux pumps and regulators.

Production system differences in carcass meat trimmings were not apparent in predominant classes of AMR. This finding was similar to another targeted shotgun metagenomic study [45] that compared conventional to raised-without-antibiotics ground beef that also found no differences in the predominant classes of resistance between the two groups (in this case 91% of the resistance gene were associated with tetracycline resistance). In a culture-based study, when conventional versus organic beef has been evaluated in terms of AMR, *Escherichia coli* and *Staphylococcus aureus* were found to harbor lower rates of AMR in organic versus conventional beef samples; although, no production differences were found in *Listeria monocytogenes* isolates [46]. While Miranda et al. did find lower AMR genes on organic meat, these differences are difficult to generalize to an ecological perspective due to the small segment of the bacterial population represented in just a few indicator organisms. The culture-based component from the same sampling as this study but separately published [19] found no differences (*P* > 0.05) between production systems in either cultured *E. coli* or tetracycline resistance *E. coli.*

Class A beta- lactamases on carcass meat trimmings were higher in CON-B samples when compared to both CON-D and ORG-D samples. Up to 63.2% of conventional dairies use cephalosporins for the treatment of mastitis [8]. Due to direct selection pressure being a known driver for AMR [47], conventional dairies would be expected to be the highest in beta-lactam resistance. As this is not the case, other environmental factors are likely to be the cause of these differences, such as cattle origin [48] or location of feeding [18]. This finding demonstrates direct selection pressure via AMD does not always correlate with higher AMR. While relatively few final meat trimming samples were collected at the end of fabrication, the relative abundance for AMR determinants at the class level were consistent with findings for carcass meat trimmings. These results may indicate that handling of beef products during fabrication of the meat for marketing was not associated with a marked increase in the abundance of resistant bacteria present on meat.

Ordination of Euclidian distances revealed that colon content resistome was altered by production system but not harvest facility. These differences were driven by a combination of low abundance AMR class differences as no class of resistance that made up more than 3% of the total relative abundance of the resistome differed between groups (such as resistance to phenicol, sulfonamides, glycopeptides, fluoroquinolones, trimethoprim, fosfomycin and rifampin). When Vikram et al. [13] compared colon content from feedlot cattle raised without antibiotics to conventional feedlot cattle using shotgun metagenomics they also did not find differences in higher abundance (tetracyclines, MLS, beta-lactams, and aminoglycosides) AMR classes. Vikram et al. [13] found abundance of genes encoding for tetracycline inactivation enzymes (TIE) higher in conventional feedlot-cattle colon content when compared to those raised without antibiotics using shotgun metagenomics, as well as several tetracycline resistance genes using qPCR, similar to the finding that TIEs were higher in CON-B than the dairy samples. Selection pressure associated with AMD exposures may explain higher relative abundance for TIE in CON-B vs. ORG-D, but tetracyclines are used in both CON-B vs. CON-D production systems [7, 8].

Resistome findings across the colon content and meat trimmings indicate that meat trimmings may have been altered to a greater extent by harvest facility, while colon content was more sensitive to the production system. These differences in resistome drivers may imply that the meat trimmings resistome is shaped, to a greater degree, by the harvest facility than fecal contamination. This does not come as a surprise as past work [49–51] has demonstrated the success of reduction of contamination on carcasses through the multiple hurtle antimicrobial intervention approach.

This study found differences in both colon content and the meat trimmings’ microbial communities by harvest facility but not production system indicating, that in this case, facility, has more of an effect on the microbiome than the production system the animal was raised in. This is in agreement with a study on the ecology of feces from calves from different farms and antibiotic treatments, which found that geographic region affected unweighted UniFrac distances while antibiotic treatment did not [52]. The disagreement between the significance of weighted and unweighted distances in the carcass meat trimmings indicates that, while the carcass meat trimmings’ microbiome between facilities had different relative abundances of bacteria present, the presence/absence of different bacteria within the samples were the same. That is, the carcass meat trimmings collected from both facilities had similar bacteria present but differed in proportions between facilities.

Facility microbiome differences are likely caused by a combination of two different drivers: geographic location and the effect of the harvest-facility environment on the meat trimmings. The first factor likely had some effect on the meat trimmings’ microbiome, but this was difficult to quantify. While the two facilities sampled were several hundred miles apart, the sources of cattle that were processed at these facilities were unknown. Nonetheless, past work has demonstrated that animal source contributes to fecal microbiome composition [48], therefore, while the geographic source of the cattle was not explicitly studied here, it may have contributed to facility differences. Another component of the meat trimmings’ microbiome differences was the harvest facility itself. Recently, there has been an increased focus on how built environments interact with organisms in these ecosystems [53]. Beef-processing environments that have unique chemical intervention systems and many employees resulting in unique commensal bacteria signatures between facilities.

While there were no resistome differences between production systems for final meat trimmings, differences were found in the microbiome analysis; although, these could be to how the facilities processed the meat trimmings. This is likely attributable to the fact that organic cow carcasses were always the first to go through fabrication at the start of the day after the facility was cleaned and sanitized. Conventional carcasses followed, with no cleaning between. As a result, there was a greater number of bacteria present at the start of the fabrication on the food contact belts for conventional carcasses than for organic carcasses. The likelihood that production differences are a result of facility sanitation system and not a pre-harvest differences is reinforced by the fact that there were no differences in the production system the cows were raised in based on the carcass meat trimmings.

A final consideration in this study is the methodology used. This paper highlights the targeted metagenomic resistome and amplicon microbiome approach while Schmidt et al. [19] reports the culture and qPCR results on the same study design and sampling scheme. The results of this paper taken with the culture and qPCR results emphasize different questions and data each method can produce. Past studies [13, 14, 54] have also compared these methodologies and very generally found that short-read metagenomics allows for a microbial ecology approach, while qPCR allows for specific detection of genes of interest, and culture allows for contextualizing AMR genes within a specific bacterium of interest and the guarantee the gene is being expressed. When approaching an issue such as AMR proliferation in the food supply, all of these approaches contribute to the public health understanding of AMR propagation. Culturing allows for the absolute quantification of pathogens with resistance to antibiotics of interest, while qPCR allows for a higher sensitively of detection and quantification of AMR genes, finally metagenomics allowing for an unbiased survey of these genes free of primer selection limitations (although PCR and metagenomics do not allow for AMR gene association with a specific pathogen). Characterization of baseline resistomes as well as genes of interest will allow further refinement in understanding what resistomes or levels of AMR genes may be considered a public health risk.

## Conclusion

The resistome of both colon content and meat trimmings was primarily composed of the same classes of resistance: tetracyclines, beta-lactams, MLS, and mechanisms that conferred resistance to more than one class of resistance. Less than half of carcass meat trimmings samples had any reads that aligned to AMR genes; of those that did, facility of harvest had more of an impact on the resistome than production system. On the other hand, the colon content resistome differed between production systems but not facility. In contrast, the microbiome of both meat trimmings and colon content was affected by facility but not production system. These differences, along with subtle difference within the mechanisms of resistance in both meat trimmings and colon content provide a descriptive baseline of the cull cattle resistome and microbiome from a culture-free metagenomic approach. Additionally, while hits to AMR genes were found in less than half of meat trimmings samples, the overall number was low and of these genes, it cannot be determined what percentage are harbored in commensal microflora versus pathogenic bacteria. While the immediate biological and public health implications of these findings are less clear, these data provide a framework understanding of the cull cattle resistome and microbiome.

## Supplementary Information

**Additional file 1: Figure S1.** As determined by ANCOM, the phyla, classes, orders, families, and genus that differed (*P* < 0.05) between facility when the microbiome of the colon content and carcass meat trimmings were compared. **Figure S2** Final meat trimmings in the Midwestern facility are colored according to the production system where the cows were raised. Alpha diversity differed (*P* < 0.05) between the two production systems as conventional cattle had higher diversity. Beta diversity also differed (*P* < 0.05) between conventionally and organically raised cows

**Additional file 2: Data 1.** Number of Individual samples within composite sample. **Data 2.** Carcass Meat Trimmings production system antimicrobial resistance determinants class log2 fold change pairwise differences. **Data 3.** Carcass Meat Trimmings facility antimicrobials resistance determinant's class log2 fold change pairwise differences. **Data 4.** Count matrix of raw reads attributed to antibiotic resistance determinant's aggregated by class of resistance. **Data 5.** Carcass Meat Trimmings production system antimicrobials resistance determinant's mechanism log2 fold change pairwise differences. **Data 6.** Carcass Meat Trimmings facility antimicrobials resistance determinant's mechanism log2 fold change pairwise differences. **Data 7.** Count matrix of raw reads attributed to antibiotic resistance determinant's aggregated by mechanism of resistance. **Data 8.** Colon content facility antimicrobials resistance determinant's class log2 fold change pairwise differences. **Data 9.** Colon content production system antimicrobials resistance determinant's class log2 fold change pairwise differences. **Data 10.** Colon content facility antimicrobials resistance determinant's mechanism log2 fold change pairwise differences. **Data 11.** Colon content production system antimicrobials resistance determinant's mechanism log2 fold change pairwise differences

## Data Availability

Raw sequence reads for all samples described in this project have been deposited the NCBI BioProject PRJNA736075.

## Abbreviations

ARG: Antimicrobial resistance gene
AMR: Antimicrobial resistance
CON-D: Conventional dairy
CON-B: Conventional beef
ORG-D: Organic dairy
