# Supplementary material for: Resistomes and microbiome of meat trimmings and colon content from culled cows raised in conventional and organic production systems
Source: Anim Microbiome. 2022 Mar 10;4:21. doi: 10.1186/s42523-022-00166-z (PMC8908682; doi:10.1186/s42523-022-00166-z)
Supplement: Supplementary file 1 — Additional file 1: Figure S1. As determined by ANCOM, the phyla, classes, orders, families, and genus that differed (P < 0.05) between facility when the microbiome of the colon content and carcass meat trimmings were compared. Figure S2 Final meat trimmings in the Midwestern facility are colored according to the production system where the cows were raised. Alpha diversity differed (P < 0.05) between the two production systems as conventional cattle had higher diversity. Beta diversity also differed (P < 0.05) between conventionally and organically raised cows [file 42523_2022_166_MOESM1_ESM.docx]

SUPPLEMENTAL FIGURES**
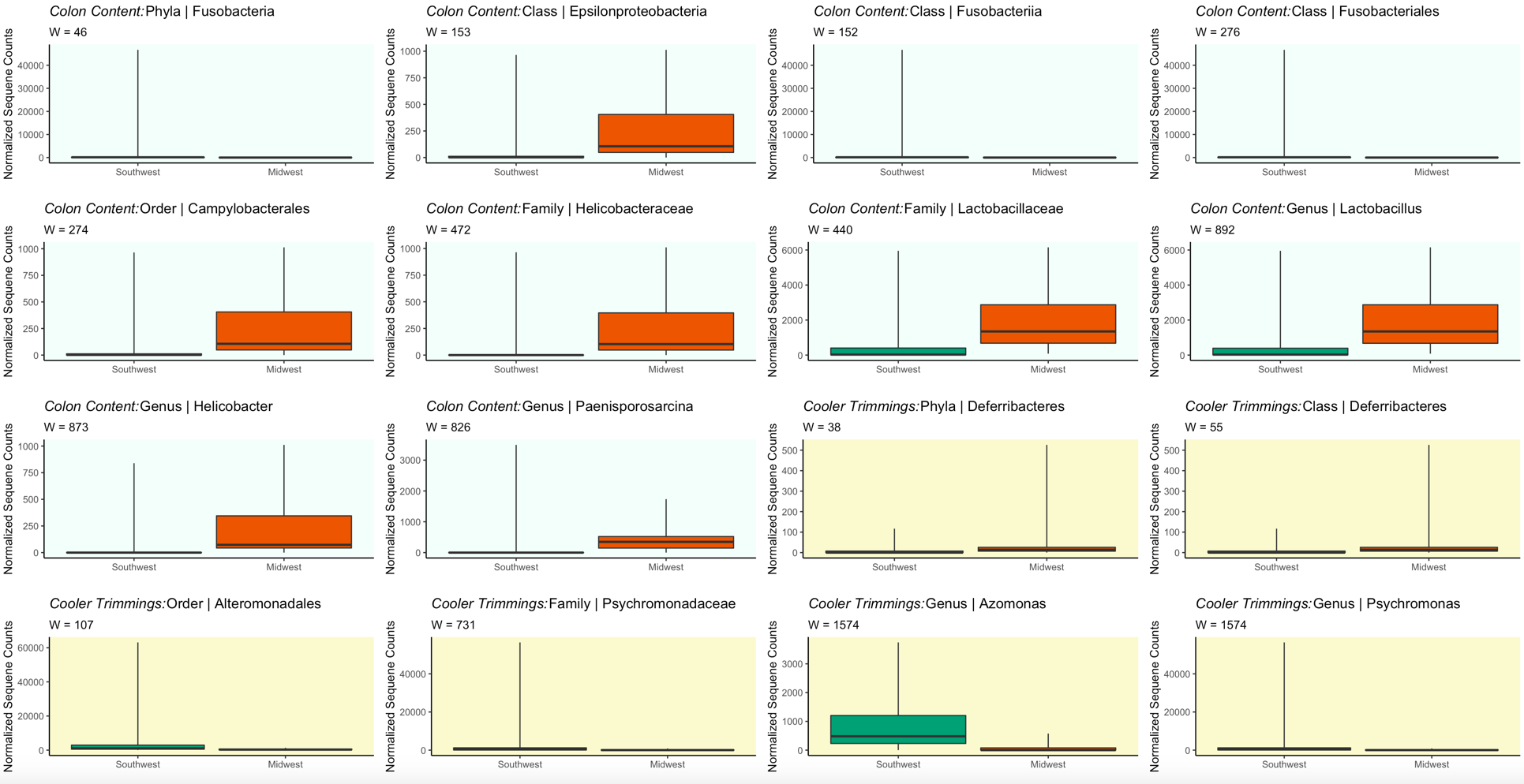
**

Supplemental Figure S1. As determined by ANCOM, the phyla, classes, orders, families, and genus that differed (*P* < 0.05) between facility when the microbiome of the colon content and carcass meat trimmings were compared.


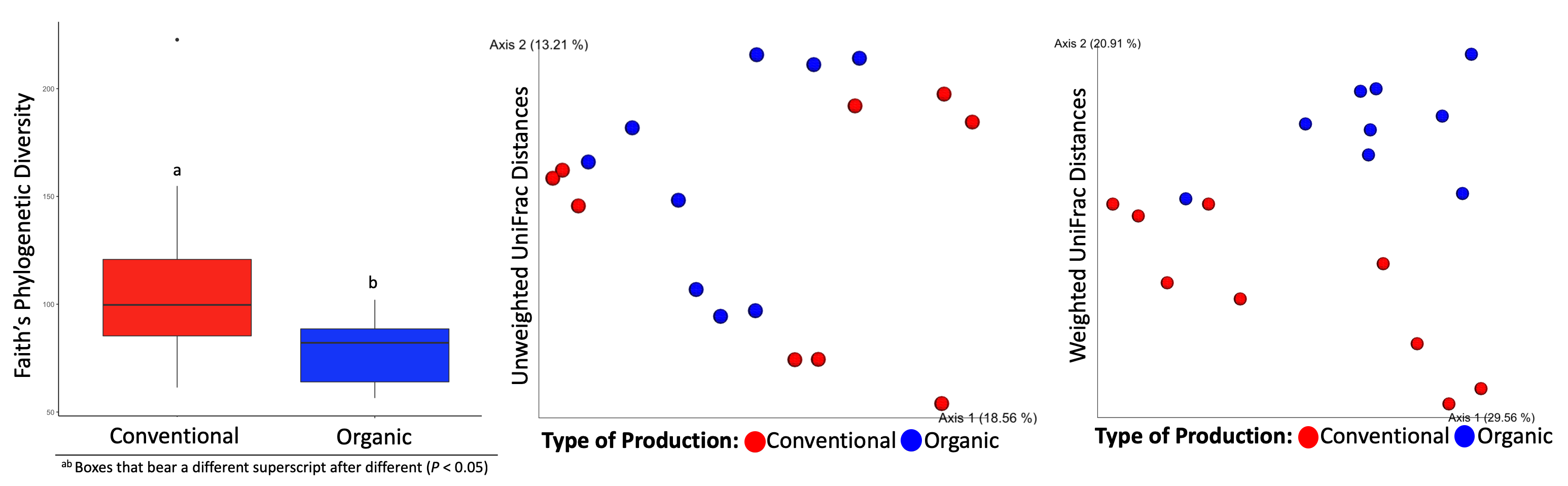


Supplemental Figure S2 Final meat trimmings in the Midwestern facility are colored according to the production system where the cows were raised. Alpha diversity differed (*P* < 0.05) between the two production systems as conventional cattle had higher diversity. Beta diversity also differed (*P* < 0.05) between conventionally and organically raised cows
